# Development and comparative evaluation of machine learning models for predicting lower extremity deep vein thrombosis in gastrointestinal cancer patients using multicenter longitudinal clinical data

**DOI:** 10.3389/fsurg.2025.1648645

**Published:** 2025-10-10

**Authors:** Jing Xu, Jue Xia, Yuan Liu, Zhiyang Jiang, Songyun Zhao, Yanfei Zhu

**Affiliations:** 1 Wuxi Medical Center of Nanjing Medical University, Wuxi, China; 2 Department of Ultrasound Medicine, The Affiliated Wuxi People's Hospital of Nanjing Medical University, Wuxi, China; 3 Department of General Surgery, Tengzhou Central People's Hospital, Jining Medical College, Shandong, China; 4 Department of General Surgery, The Affiliated Wuxi People's Hospital of Nanjing Medical University, Wuxi, China; 5 Department of Plastic Surgery, The Affiliated Friendship Plastic Surgery Hospital of Nanjing Medical University, Nanjing, China; 6 Department of Plastic Surgery, The First Affiliated Hospital of Wenzhou Medical University, Wenzhou, China

**Keywords:** deep vein thrombosis, gastrointestinal neoplasm, machine learning, XGBoost, risk factor

## Abstract

**Background:**

Lower extremity deep vein thrombosis (DVT) represents a prevalent and formidable complication among patients with gastrointestinal malignancies, exerting a profound impact on both prognosis and quality of life. Owing to its intricate pathogenesis, the development of a precise risk prediction model is imperative for advancing clinical strategies in prevention and therapeutic intervention.

**Methods:**

This retrospective study enrolled patients with gastrointestinal malignancies using multicenter, longitudinal clinical data obtained from three tertiary medical centers between 2020 and 2024. A total of 34 variables were extracted, encompassing demographic profiles, clinical parameters, tumor-specific characteristics, and laboratory indices. To identify independent predictors of DVT, both univariate and multivariate analyses were initially performed. Four machine learning algorithms—Extreme Gradient Boosting (XGBoost), Random Forest (RF), Support Vector Machine (SVM), and k-Nearest Neighbors (KNN)—were subsequently constructed to predict DVT risk. Model performance was rigorously assessed through receiver operating characteristic (ROC) curves, calibration plots, Brier scores, and decision curve analysis (DCA). Internal validation was conducted via ten-fold cross-validation, while an independent external cohort was employed to evaluate model generalizability. To elucidate the underlying predictive mechanisms, SHapley Additive exPlanations (SHAP) analysis was carried out.

**Results:**

Through a combination of univariate and multivariate analyses alongside four machine learning algorithms, surgery, prolonged immobilization, central venous catheterization, radiotherapy, distant metastasis, and chemotherapy emerged as significant high-risk factors for DVT. All four predictive models exhibited robust performance, with the XGBoost model demonstrating superior discrimination, calibration, and clinical utility. Findings from the external validation cohort further substantiated its stability and generalizability. SHAP analysis illuminated the relative contributions and directional influences of pivotal variables within the predictive framework.

**Conclusion:**

Machine learning models derived from multicenter, longitudinal clinical datasets offer robust predictive capabilities for assessing DVT risk in patients with gastrointestinal malignancies. These models furnish clinicians with individualized risk stratification tools, facilitating the refinement of preventive strategies and the enhancement of clinical decision-making, ultimately contributing to improved patient management.

## Introduction

Gastrointestinal malignancies rank among the most lethal cancers globally. Driven by a rapidly aging population and the pervasive adoption of deleterious lifestyle behaviors, the incidence of these tumors continues to climb, constituting a substantial fraction of the global oncological burden. Despite notable progress in early detection and surgical interventions in recent years, the majority of cases are diagnosed at intermediate or advanced stages, often accompanied by multiple comorbidities that significantly undermine clinical outcomes (1–5).

Lower extremity DVT is a frequent and formidable complication in patients with gastrointestinal tumors, marked by a high incidence and significant risks of disability and mortality (6–8). Neoplastic processes themselves foster a hypercoagulable milieu through the secretion of procoagulant factors such as tissue factor and tumor-derived microparticles, which activate the coagulation cascade. Moreover, chronic tumor-associated inflammation, endothelial injury inflicted by malignant cells, and disruption of the immune microenvironment synergistically promote thrombus formation (9–11).

The consequences of thrombosis extend well beyond localized symptoms such as limb edema, pain, and impaired mobility. Thrombus dislodgement can precipitate pulmonary embolism (PE)—a life-threatening emergency. These complications not only prolong hospitalization and elevate the risk of bleeding associated with anticoagulant therapy but may also interrupt or even preclude standardized oncologic treatments, thereby compromising disease control and overall survival (12–14). A growing body of evidence (15–19) underscores that cancer patients who develop venous thromboembolism (VTE) face a markedly heightened risk of mortality within one year, rendering VTE a principal cause of non-cancer-related death in this population.

Traditionally, clinicians have relied on experiential judgment or risk stratification tools such as the Caprini and Khorana scores to assess thrombotic risk. While these instruments provide a degree of guidance, they are constrained by inherent subjectivity, limited generalizability, and suboptimal accuracy in detecting tumor-associated thrombosis (20, 21). Recently, statistical approaches like logistic regression have been employed to enhance predictive objectivity and quantification; nevertheless, these methods falter when confronted with high-dimensional datasets, nonlinear relationships, and intricate variable interactions, limiting their applicability in complex clinical landscapes.

Against this backdrop, the present study seeks to leverage multiple sophisticated machine learning algorithms to assimilate multidimensional clinical data and develop a predictive model for delineating high-risk factors of lower extremity venous thrombosis in patients with gastrointestinal malignancies. This model aspires to elevate the precision and efficiency of high-risk patient identification, thereby furnishing robust empirical support and a solid foundation for individualized prophylactic interventions.

## Materials and methods

### Study subjects

This study utilized clinical data sourced from the databases of Wuxi People's Hospital affiliated with Nanjing Medical University, Wuxi Second People's Hospital, and Tengzhou Central People's Hospital. The clinical data and samples analyzed in this study were collected from January 1, 2020, to January 31, 2024, and the datasets were accessed for research purposes on January 31, 2024. Inclusion criteria encompassed: (1) patients with pathologically and radiologically confirmed gastrointestinal malignancies, including esophageal, gastric, small intestinal, colorectal, pancreatic cancers, cholangiocarcinoma, and hepatocellular carcinoma; (2) age ≥18 years; and (3) completion of lower extremity venous ultrasound screening during hospitalization. Exclusion criteria were as follows: (1) presence of other malignancies; (2) prior history of lower extremity DVT preceding the diagnosis of gastrointestinal tumors; (3) anticoagulant therapy exceeding two weeks, including agents such as warfarin, rivaroxaban, apixaban, and heparin; (4) severe hepatic or renal insufficiency or coagulation disorders, including congenital conditions (e.g., hemophilia) or acute disseminated intravascular coagulation (DIC); (5) pregnancy or lactation; (6) mortality within 30 days of admission; and (7) incomplete clinical data or loss to follow-up. All patients were monitored for a minimum of six months postoperatively. This investigation received ethical approval from the Institutional Review Boards of Wuxi People's Hospital, Wuxi Second People's Hospital, and Tengzhou Central People's Hospital (Approval No. 2025-37). We have strictly adhered to the guidelines of the TRIPOD + AI statement (https://www.tripod-statement.org/).

### Study design and data collection

This study encompassed a total of 34 clinical variables spanning multiple domains to facilitate a comprehensive evaluation of lower extremity DVT risk. The variables were systematically classified as follows: firstly, demographic attributes including sex, age, smoking status, alcohol consumption, and body mass index (BMI); secondly, baseline clinical indices such as the American Society of Anesthesiologists (ASA) score, Nutritional Risk Screening 2002 (NRS2002) score, history of blood transfusion, venous catheterization history, and duration of immobilization; thirdly, comorbidities encompassing anemia, coronary artery disease, intestinal obstruction, chronic obstructive pulmonary disease (COPD), diabetes mellitus, hypertension, and hyperlipidemia; fourthly, tumor-specific features comprising tumor type, maximal diameter, lesion multiplicity, regional lymph node involvement, distant metastasis, perineural invasion, and receipt of surgery, chemotherapy, or radiotherapy; and finally, laboratory biomarkers including serum albumin, carcinoembryonic antigen (CEA), carbohydrate antigen 19-9 (CA19-9), procalcitonin (PCT), C-reactive protein (CRP), neutrophil-to-lymphocyte ratio (NLR), and serum amyloid A (SAA). The principal endpoint of this investigation was the incidence of lower extremity deep vein thrombosis.

### Missing data handling and data scaling

Variables with a missing rate below 5% were classified as exhibiting low missingness, whereas those with a missing rate between 5% and 30% were deemed to have moderate to high missingness. Two complementary strategies were employed to address missing data. For variables with low missingness, simple imputation was applied: median imputation for continuous variables and mode imputation (most frequent category) for categorical variables. This approach, restricted to minimal missingness, aimed to preserve sample integrity and was subsequently evaluated against multiple imputation outcomes in sensitivity analyses.

For variables with moderate to high missingness, multiple imputation was performed. Binary variables (e.g., sex, presence of comorbidities) were imputed using logistic regression models, in which the probability distribution of missing values was estimated from available predictors, followed by stochastic sampling to preserve intrinsic inter-variable correlations. For multicategorical variables (e.g., tumor location, staging), multinomial logistic regression was employed, simultaneously estimating the probability of each mutually exclusive category and imputing missing entries through probabilistic sampling. This method maintained the original distributional structure of the data, mitigated bias, and improved the plausibility of imputations, thereby enhancing the predictive robustness of subsequent models.

Continuous variables were discretized into binary or multicategorical forms guided by clinical expertise, while categorical variables underwent one-hot encoding to ensure accurate model recognition of categorical information.

### Diagnosis of DVT and definition of associated factors

Lower extremity DVT denotes the pathological coagulation of blood within the deep venous system of the lower limbs—including the peroneal, posterior tibial, popliteal, femoral, and iliac veins—culminating in thrombus formation and vascular occlusion (22–24). In this investigation, the initial diagnosis predominantly hinged on Doppler ultrasound, with diagnostic criteria comprising partial or complete incompressibility of the vein (under physiological conditions, veins collapse entirely under probe pressure; failure to do so indicates thrombus presence), aberrant blood flow signals (color Doppler revealing diminished or interrupted flow), direct visualization of thrombotic echoes on grayscale imaging, and abnormal pulse Doppler waveforms characterized by reduced or absent flow velocity. In instances where ultrasonographic findings were equivocal—particularly when evaluating deep or pelvic veins such as the iliac vein—or where clinical suspicion remained high despite negative ultrasound, venography was employed as an adjunct. This technique, involving intravascular contrast administration, affords three-dimensional visualization, enabling precise delineation of thrombus burden and localization, thereby enhancing diagnostic fidelity. We ensure that all DVT events were confirmed by imaging, guaranteeing the consistency of diagnostic criteria and the accuracy of the results.

### Development and evaluation of predictive models for machine learning algorithms

This study employed SPSS and R software to construct and systematically evaluate clinical prediction models through the following steps:

#### Data preprocessing

The study population comprised patients with gastrointestinal tumors treated from January 2020 to January 2024 at Wuxi People's Hospital and Wuxi Second People's Hospital, forming the internal validation cohort. Concurrently, patients from Tengzhou Central People's Hospital during the same period constituted the external validation cohort to assess model generalizability. Within the internal cohort, stratified random sampling divided data into a training set and testing set at a 7:3 ratio, enhancing the model's capacity to detect minority events such as DVT, thereby mitigating bias toward the majority class and improving clinical applicability and predictive performance.

#### Feature selection

A systematic statistical analysis of candidate variables was performed on the internal cohort to identify clinical features significantly associated with DVT. Univariate analysis employed chi-square tests for categorical variables and independent samples *t*-tests for continuous variables to screen potential risk factors (*P* < 0.05). Significant variables were then included in a multivariate logistic regression model to adjust for confounding and identify independent predictors, with adjusted regression coefficients and 95% confidence intervals quantifying association strength. Complementing traditional statistics, four classical machine learning algorithms—XGBoost, RF, SVM, and KNN—were used to evaluate variable importance and inter-algorithm differences. Cross-validation of feature rankings across models enabled selection of the top ten consistently important variables as key predictors, thereby enhancing the robustness and interpretability of the feature screening process. Model Construction and Evaluation: The selected features were integrated into four machine learning models—SVM, RF, XGBoost, and KNN—to develop DVT risk prediction models. Model performance was assessed through discrimination, calibration, and clinical utility. Discrimination was measured by ROC curves and AUC metrics to evaluate the ability to distinguish between DVT and non-DVT cases. Calibration was evaluated by constructing calibration curves to compare the concordance between predicted probabilities and observed event rates, supplemented by the Brier score as a quantitative measure of probabilistic accuracy. In these curves, the *x*-axis (mean predicted value) denotes the average model-estimated probability of an event (e.g., DVT) within a given subgroup, reflecting its anticipated risk, while the *y*-axis (fraction of positives) represents the corresponding empirical event rate, i.e., the true incidence of DVT within that subgroup. This graphical assessment captures the degree to which predicted probabilities align with actual outcomes. The ideal calibration curve coincides with the 45° diagonal, indicating perfect agreement between predicted and observed rates. In this study, calibration curves were generated for the XGBoost, RF, SVM, and KNN models to assess their probability estimation fidelity. Samples were stratified into equally sized risk groups (e.g., deciles) according to predicted probabilities; for each group, the mean predicted risk and the observed incidence were computed, and both scatter plots and fitted calibration lines were produced. Clinical utility was appraised using decision curve analysis (DCA), which plots net benefit across a continuum of clinical risk thresholds (0–1), benchmarked against treat-all and treat-none strategies, thereby identifying threshold intervals in which the model confers superior clinical advantage. Guided by expert clinical consensus, we selected a threshold range of 0.1–0.6, corresponding to commonly adopted cut-offs for DVT prophylaxis that strike a balance between proactive prevention and avoidance of unnecessary intervention. To improve reliability and minimize bias from data splitting, 10-fold cross-validation was applied in the internal cohort, iteratively training on nine folds and validating on the remaining fold. Performance metrics, including accuracy, AUC, and Brier score, were averaged across folds, providing robust estimates of model stability and generalization. In this study, hyperparameter optimization was performed using a grid search strategy. This method exhaustively evaluates all possible parameter combinations within a predefined search space, identifying the configuration that yields optimal performance on the validation set through cross-validation. By systematically traversing the parameter grid, grid search ensures that no potentially superior configuration is overlooked, making it particularly well-suited for parameter spaces of moderate dimensionality. Although computationally intensive, this approach offers robust and reproducible hyperparameter selection, thereby enhancing the model's generalizability and predictive accuracy. Using this framework, we comprehensively compared the predictive performance of four machine learning models for DVT risk assessment and subsequently selected the XGBoost model for further refinement. In training the XGBoost model, particular attention was given to tuning regularization-related parameters. L1 regularization (alpha) imposes an absolute penalty on feature weights, promoting sparsity and implicit feature selection; L2 regularization (lambda) applies a squared penalty to constrain weight magnitude, mitigating overfitting; the maximum tree depth (max_depth) was limited to prevent overly complex tree structures; the minimum child weight (min_child_weight) was set to define the minimal sum of instance weights required for a node split; and the learning rate (eta) was adjusted to incrementally reduce the contribution of individual trees, thereby smoothing the learning process. Collectively, these measures preserved the model's capacity to capture intricate data patterns while reducing overfitting risk, ultimately improving its stability and generalizability across both internal and external validation cohorts.

#### External validation

The optimal model, with parameters fixed during internal training, was applied to the external validation cohort from Tengzhou Central People's Hospital. Performance metrics were computed and compared with internal results to assess generalizability and clinical applicability.

#### Construction of confusion matrices

Confusion matrix plots were generated for the XGBoost model across the internal test set, internal validation set, external test set, and external validation set. These matrices provide an intuitive visualization of classification performance, delineating the exact counts of true positives, false positives, true negatives, and false negatives. Such representation enables a more granular assessment of the model's sensitivity and specificity under varying data conditions.

#### Retrospective evaluation of the Khorana score

A supplementary retrospective analysis was undertaken to assess the predictive utility of the Khorana score in estimating lower extremity DVT risk among the study cohort. For each patient, a risk score was computed in accordance with the Khorana scoring system, which assigns weighted points based on tumor type, platelet count, hemoglobin concentration, white blood cell count, and body mass index. Predictive performance was quantified using ROC curve analysis, with the AUC and corresponding 95% CI calculated. The AUC of the Khorana score was subsequently compared with that of the best-performing machine learning model identified in this study, thereby corroborating the superior predictive accuracy of our model.

#### Model interpretation

To elucidate model decision-making, SHAP analysis was conducted. SHAP calculates each feature's marginal contribution—or “Shapley value”—across all possible feature subsets, fairly attributing feature impact on predictions. SHAP values indicate whether a feature increases or decreases predicted risk. Visualization included SHAP summary plots, showing the distribution and directional influence of each feature's SHAP values across all samples, with color gradients reflecting original feature values to reveal key risk factors and effect patterns. Additionally, single-sample SHAP force plots illustrated individualized explanations, demonstrating how each feature's contribution shifts the prediction from a baseline risk to the final predicted value, highlighting personalized risk drivers or mitigators.

## Results

### Basic clinical information of the patient

**Figure 1:**
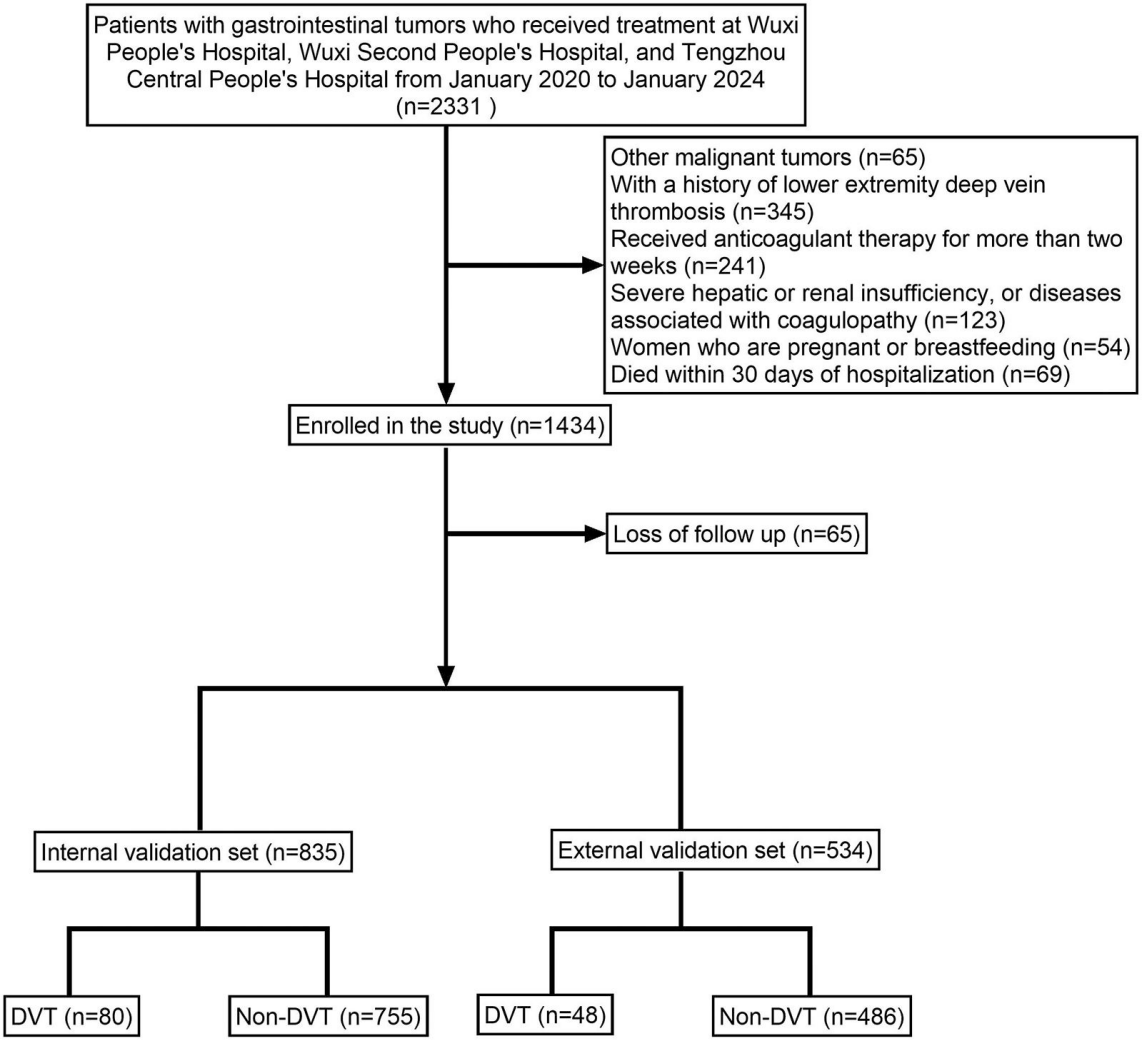
Illustrates the patient enrollment flowchart, clearly depicting the sample selection process.

**Figure 2:**
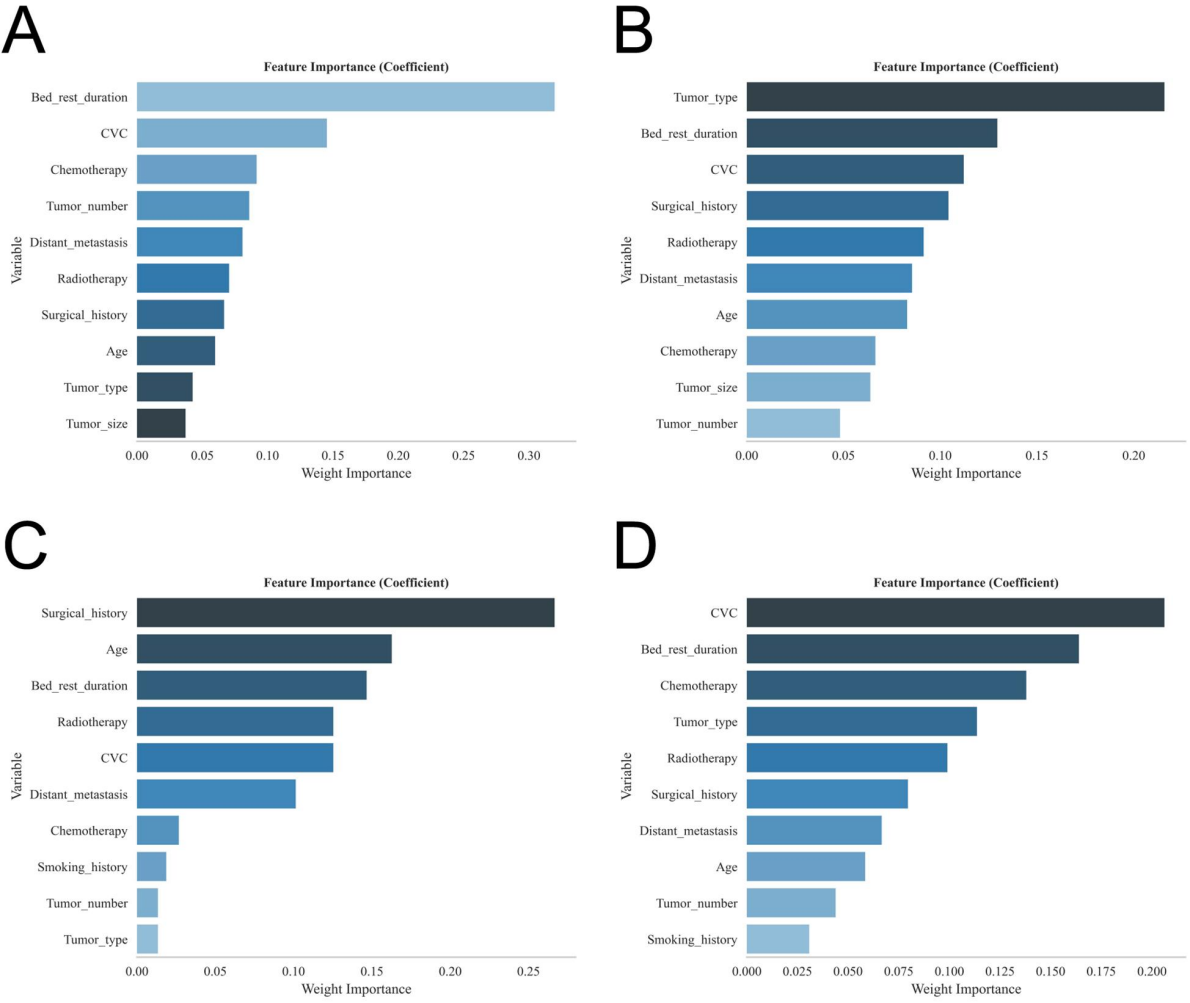
Shows the feature importance rankings for each of the four models: **(A)** XGBoost; **(B)** RF; **(C)** SVM; and **(D)** KNN.

A total of 1,369 patients with gastrointestinal tumors were enrolled in this study (Figure 1), of whom 128 patients (9.35%) developed lower extremity venous thrombosis. The internal dataset comprised 835 patients with gastrointestinal malignancies, including 80 cases of DVT, while the external dataset included 534 patients, of whom 48 had DVT. A comparison of their clinical characteristics is presented in Table 1. Univariate and multivariate analyses identified distant metastasis, duration of bed rest, central venous catheterization, hypertension, radiotherapy, chemotherapy, surgical treatment, and advanced age as independent risk factors for lower extremity venous thrombosis (*P* < 0.05) (Table 2). Feature selection using the XGBoost, RF, SVM, and KNN algorithms consistently underscored distant metastasis, duration of bed rest, central venous catheterization, radiotherapy, chemotherapy, and surgical treatment as key predictors influencing the occurrence of lower extremity venous thrombosis (Figures 2A–D). The original dataset utilized in this study is provided in Supplementary Table S1.

**Table 1:** Comparison of features between the internal and external datasets.

Variables		All (*N* = 1369)	Internal dataset (*N* = 835)	External dataset (*N* = 534)	*P*-value
Sex	Female	823 (60.117%)	555 (66.467%)	268 (50.187%)	<0.001
	Male	546 (39.883%)	280 (33.533%)	266 (49.813%)	
Age	<65	932 (68.079%)	662 (79.281%)	270 (50.562%)	<0.001
	≥65	437 (31.921%)	173 (20.719%)	264 (49.438%)	
BMI	<25 kg/m^2^	991 (72.389%)	685 (82.036%)	306 (57.303%)	<0.001
	≥25 kg/m^2^	378 (27.611%)	150 (17.964%)	228 (42.697%)	
ASA	<3	657 (47.991%)	396 (47.425%)	261 (48.876%)	0.639
	≥3	712 (52.009%)	439 (52.575%)	273 (51.124%)	
Drinking history	No	640 (46.749%)	380 (45.509%)	260 (48.689%)	0.274
	Yes	729 (53.251%)	455 (54.491%)	274 (51.311%)	
Smoking history	No	691 (50.475%)	417 (49.940%)	274 (51.311%)	0.66
	Yes	678 (49.525%)	418 (50.060%)	260 (48.689%)	
Surgical history	No	941 (68.736%)	634 (75.928%)	307 (57.491%)	<0.001
	Yes	428 (31.264%)	201 (24.072%)	227 (42.509%)	
Chemotherapy	No	990 (72.316%)	611 (73.174%)	379 (70.974%)	0.409
	Yes	379 (27.684%)	224 (26.826%)	155 (29.026%)	
Radiotherapy	No	949 (69.321%)	564 (67.545%)	385 (72.097%)	0.085
	Yes	420 (30.679%)	271 (32.455%)	149 (27.903%)	
ALB	≥30 g/L	809 (59.094%)	570 (68.263%)	239 (44.757%)	<0.001
	<30 g/L	560 (40.906%)	265 (31.737%)	295 (55.243%)	
CEA level	<5 ng/ml	872 (63.696%)	606 (72.575%)	266 (49.813%)	<0.001
	≥5 ng/ml	497 (36.304%)	229 (27.425%)	268 (50.187%)	
CA199 level	<37 U/ml	869 (63.477%)	619 (74.132%)	250 (46.816%)	<0.001
	≥37 U/ml	500 (36.523%)	216 (25.868%)	284 (53.184%)	
NRS2002 score	<3	853 (62.308%)	570 (68.263%)	283 (52.996%)	<0.001
	≥3	516 (37.692%)	265 (31.737%)	251 (47.004%)	
Anemia	No	862 (62.966%)	589 (70.539%)	273 (51.124%)	<0.001
	Yes	507 (37.034%)	246 (29.461%)	261 (48.876%)	
Ileus	No	853 (62.308%)	573 (68.623%)	280 (52.434%)	<0.001
	Yes	516 (37.692%)	262 (31.377%)	254 (47.566%)	
CHD	No	929 (67.860%)	662 (79.281%)	267 (50.000%)	<0.001
	Yes	440 (32.140%)	173 (20.719%)	267 (50.000%)	
COPD	No	939 (68.590%)	673 (80.599%)	266 (49.813%)	<0.001
	Yes	430 (31.410%)	162 (19.401%)	268 (50.187%)	
Diabetes	No	822 (60.044%)	549 (65.749%)	273 (51.124%)	<0.001
	Yes	547 (39.956%)	286 (34.251%)	261 (48.876%)	
Hyperlipidemia	No	839 (61.286%)	582 (69.701%)	257 (48.127%)	<0.001
	Yes	530 (38.714%)	253 (30.299%)	277 (51.873%)	
Hypertension	No	943 (68.882%)	669 (80.120%)	274 (51.311%)	<0.001
	Yes	426 (31.118%)	166 (19.880%)	260 (48.689%)	
Blood transfusion	No	830 (60.628%)	561 (67.186%)	269 (50.375%)	<0.001
	Yes	539 (39.372%)	274 (32.814%)	265 (49.625%)	
CVC	No	964 (70.416%)	611 (73.174%)	353 (66.105%)	0.006
	Yes	405 (29.584%)	224 (26.826%)	181 (33.895%)	
Bed rest duration	<3	960 (70.124%)	598 (71.617%)	362 (67.790%)	0.148
	≥3	409 (29.876%)	237 (28.383%)	172 (32.210%)	
Tumor type	Pancreatic cancer	115 (8.400%)	77 (9.222%)	38 (7.116%)	<0.001
	Esophageal cancer	71 (5.186%)	61 (7.305%)	10 (1.873%)	
	Gastric cancer	496 (36.231%)	272 (32.575%)	224 (41.948%)	
	Small intestine tumor	247 (18.042%)	143 (17.126%)	104 (19.476%)	
	Colorectal cancer	113 (8.254%)	74 (8.862%)	39 (7.303%)	
	Hepatocellular carcinoma	148 (10.811%)	107 (12.814%)	41 (7.678%)	
	Cholangiocarcinoma	179 (13.075%)	101 (12.096%)	78 (14.607%)	
Tumor number	<2	940 (68.663%)	693 (82.994%)	247 (46.255%)	<0.001
	≥2	429 (31.337%)	142 (17.006%)	287 (53.745%)	
Tumor size	<5 cm	834 (60.920%)	557 (66.707%)	277 (51.873%)	<0.001
	≥5 cm	535 (39.080%)	278 (33.293%)	257 (48.127%)	
Lymphatic metastasis	No	905 (66.107%)	621 (74.371%)	284 (53.184%)	<0.001
	Yes	464 (33.893%)	214 (25.629%)	250 (46.816%)	
Distant metastasis	No	967 (70.636%)	613 (73.413%)	354 (66.292%)	0.006
	Yes	402 (29.364%)	222 (26.587%)	180 (33.708%)	
PNI	No	1,016 (74.215%)	744 (89.102%)	272 (50.936%)	<0.001
	Yes	353 (25.785%)	91 (10.898%)	262 (49.064%)	
PCT level	<0.05 ng/ml	970 (70.855%)	683 (81.796%)	287 (53.745%)	<0.001
	≥0.05 ng/ml	399 (29.145%)	152 (18.204%)	247 (46.255%)	
CRP level	<10 mg/L	909 (66.399%)	651 (77.964%)	258 (48.315%)	<0.001
	≥10 mg/L	460 (33.601%)	184 (22.036%)	276 (51.685%)	
SAA level	<10 mg/L	910 (66.472%)	644 (77.126%)	266 (49.813%)	<0.001
	≥10 mg/L	459 (33.528%)	191 (22.874%)	268 (50.187%)	
NLR	<3	873 (63.769%)	612 (73.293%)	261 (48.876%)	<0.001
	≥3	496 (36.231%)	223 (26.707%)	273 (51.124%)	
DVT	No	1,241 (90.650%)	755 (90.419%)	486 (91.011%)	0.786
	Yes	128 (9.350%)	80 (9.581%)	48 (8.989%)	

OR, odds ratio; CI, confidence interval; BMI, body mass index; ASA, The American Society of Anesthesiologists; ALB, albumin; PCT, procalcitonin; CRP, C-reactive protein; NLR, neutrophil to lymphocyte ratio; SAA, serum amyloid A; NRS2002, nutrition risk screening 2002; CVC, Central venous catheter; PNI, Perineural invasion; CHD, Coronary heart disease; COPD, Chronic obstructive pulmonary disease.

**Table 2:** Presents the results of univariate and multivariate analyses of variables associated with DVT.

Variables			Univariate analysis			Multivariate analysis		
			OR	95% CI	*P*-value	OR	95% CI	*P*-value
Sex	Female	555	Reference			Reference		
	Male	280	4.039	[2.496, 6.538]	<0.001	1.854	[0.879, 3.959]	0.106
Age	<65	662	Reference			Reference		
	≥65	173	3.477	[2.155, 5.613]	<0.001	2.532	[1.127, 5.727]	0.025
BMI	<25 kg/m^2^	685	Reference			Reference		
	≥25 kg/m^2^	150	1.995	[1.186, 3.357]	0.009	0.831	[0.354, 1.896]	0.665
ASA	<3	396	Reference					
	≥3	439	0.943	[0.595, 1.495]	0.803			
Drinking history	No	380	Reference					
	Yes	455	1.359	[0.848, 2.178]	0.203			
Smoking history	No	417	Reference					
	Yes	418	0.893	[0.563, 1.416]	0.63			
Surgical history	No	634	Reference			Reference		
	Yes	201	7.093	[4.347, 11.574]	<0.001	3.787	[1.888, 7.746]	<0.001
Chemotherapy	No	611	Reference			Reference		
	Yes	224	0.5	[0.270, 0.925]	0.027	0.223	[0.086, 0.533]	0.001
Radiotherapy	No	564	Reference			Reference		
	Yes	271	4.277	[2.641, 6.926]	<0.001	2.793	[1.417, 5.617]	0.003
ALB	≥30 g/L	570	Reference			Reference		
	<30 g/L	265	9.366	[5.410, 16.216]	<0.001	2.185	[0.966, 5.066]	0.063
CEA level	<5 ng/ml	606	Reference					
	≥5 ng/ml	229	0.583	[0.326, 1.045]	0.07			
CA199 level	<37 U/ml	619	Reference					
	≥37 U/ml	216	0.636	[0.355, 1.141]	0.129			
NRS2002 score	<3	570	Reference					
	≥3	265	1.177	[0.725, 1.910]	0.51			
Anemia	No	589	Reference					
	Yes	246	1.171	[0.715, 1.918]	0.531			
Ileus	No	573	Reference			Reference		
	Yes	262	3.011	[1.886, 4.806]	<0.001	1.647	[0.827, 3.258]	0.152
CHD	No	662	Reference					
	Yes	173	1.312	[0.767, 2.242]	0.322			
COPD	No	673	Reference					
	Yes	162	1.333	[0.772, 2.302]	0.302			
Diabetes	No	549	Reference			Reference		
	Yes	286	3.075	[1.920, 4.924]	<0.001	1.869	[0.845, 4.14]	0.121
Hyperlipidemia	No	582	Reference					
	Yes	253	0.642	[0.372, 1.110]	0.113			
Hypertension	No	669	Reference			Reference		
	Yes	166	9.787	[5.953, 16.091]	<0.001	5.966	[2.984, 12.215]	<0.001
Blood transfusion	No	561	Reference			Reference		
	Yes	274	2.636	[1.654, 4.201]	<0.001	1.173	[0.524, 2.626]	0.696
CVC	No	611	Reference			Reference		
	Yes	224	6.704	[4.092, 10.984]	<0.001	6.7	[3.263, 14.183]	<0.001
Bed rest duration	<3	598	Reference			Reference		
	≥3	237	11.416	[6.578, 19.813]	<0.001	2.949	[1.335, 6.661]	0.008
Tumor type	Pancreatic cancer	77	Reference					
	Esophageal cancer	61	1.534	[0.487, 4.827]	0.464			
	Gastric cancer	272	1.093	[0.429, 2.788]	0.852			
	Small intestine tumor	143	1.284	[0.473, 3.488]	0.624			
	Colorectal cancer	74	1.434	[0.473, 4.354]	0.524			
	Hepatocellular carcinoma	107	0.703	[0.218, 2.269]	0.555			
	Cholangiocarcinoma	101	2.227	[0.828, 5.993]	0.113			
Tumor number	<2	693	Reference					
	≥2	142	0.759	[0.391, 1.475]	0.416			
Tumor size	<5 cm	557	Reference			Reference		
	≥5 cm	278	3.852	[2.387, 6.217]	<0.001	0.941	[0.425, 2.064]	0.879
Lymphatic metastasis	No	621	Reference					
	Yes	214	1.112	[0.663, 1.866]	0.687			
Distant metastasis	No	613	Reference			Reference		
	Yes	222	4.199	[2.617, 6.736]	<0.001	2.12	[1.058, 4.232]	0.033
PNI	No	744	Reference					
	Yes	91	1.04	[0.501, 2.160]	0.915			
PCT level	<0.05 ng/ml	683	Reference					
	≥0.05 ng/ml	152	1.041	[0.576, 1.880]	0.894			
CRP level	<10 mg/L	651	Reference			Reference		
	≥10 mg/L	184	1.94	[1.182, 3.185]	0.009	1.188	[0.556, 2.472]	0.649
SAA level	<10 mg/L	644	Reference					
	≥10 mg/L	191	0.829	[0.467, 1.470]	0.52			
NLR	<3	612	Reference					
	≥3	223	1.045	[0.624, 1.753]	0.866			

OR, odds ratio; CI, confidence interval; BMI, body mass index; ASA, The American Society of Anesthesiologists; ALB, albumin; PCT, procalcitonin; CRP, C-reactive protein; NLR, neutrophil to lymphocyte ratio; SAA, serum amyloid A; NRS2002, nutrition risk screening 2002; CVC, central venous catheter; PNI, perineural invasion; CHD, coronary heart disease; COPD, chronic obstructive pulmonary disease.

### Model building and evaluation

**Figure 3:**
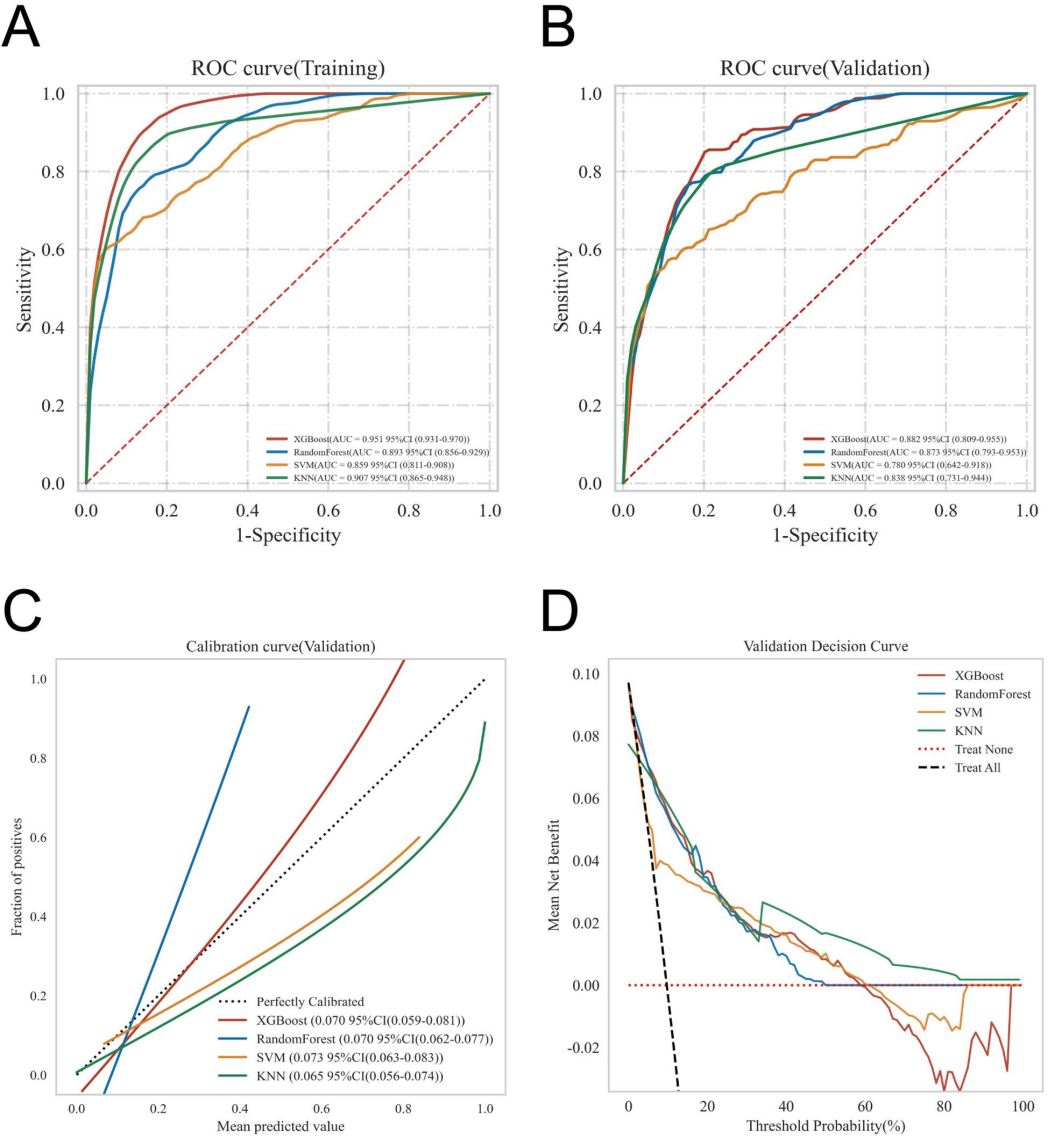
Provides a comprehensive evaluation of the predictive performance of the four models, including: **(A)** ROC curves for the training set; **(B)** ROC curves for the validation set; **(C)** calibration curves, where the 45° dashed line represents ideal agreement between predicted and observed outcomes—curves closer to this line indicate better calibration; and **(D)** DCA, with the red curve indicating the net benefit of the model across varying risk thresholds. The intersections between the red curve and the “All” and “None” strategies define the risk threshold ranges where the model confers clinical benefit.

**Figure 4:**
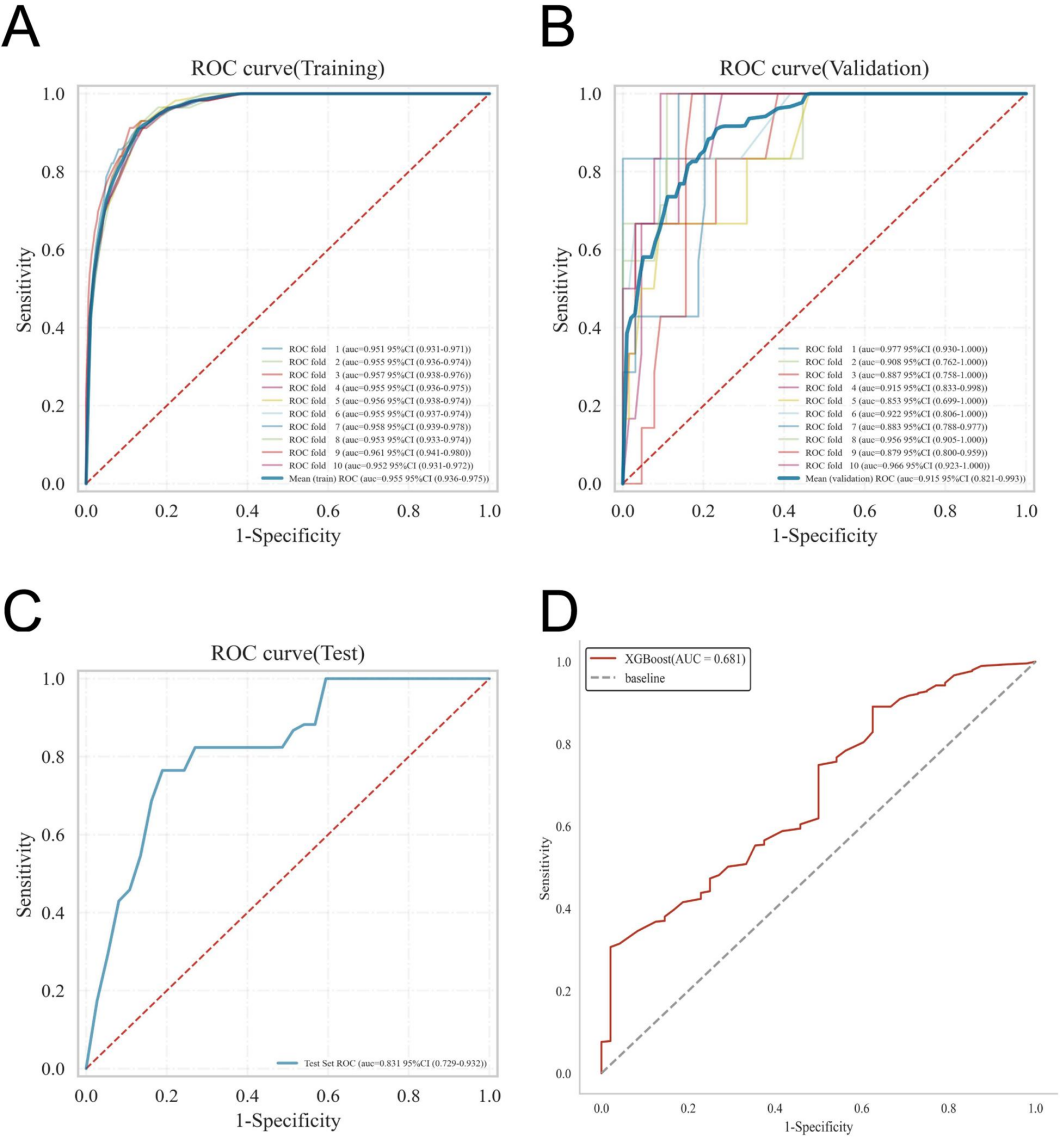
Details the internal and external validation results of the XGBoost model, including: **(A)** ROC curve in the training set; **(B)** ROC curve in the validation set; **(C)** ROC curve in the testing set; and **(D)** ROC curve in the external validation cohort.

**Figure 5:**
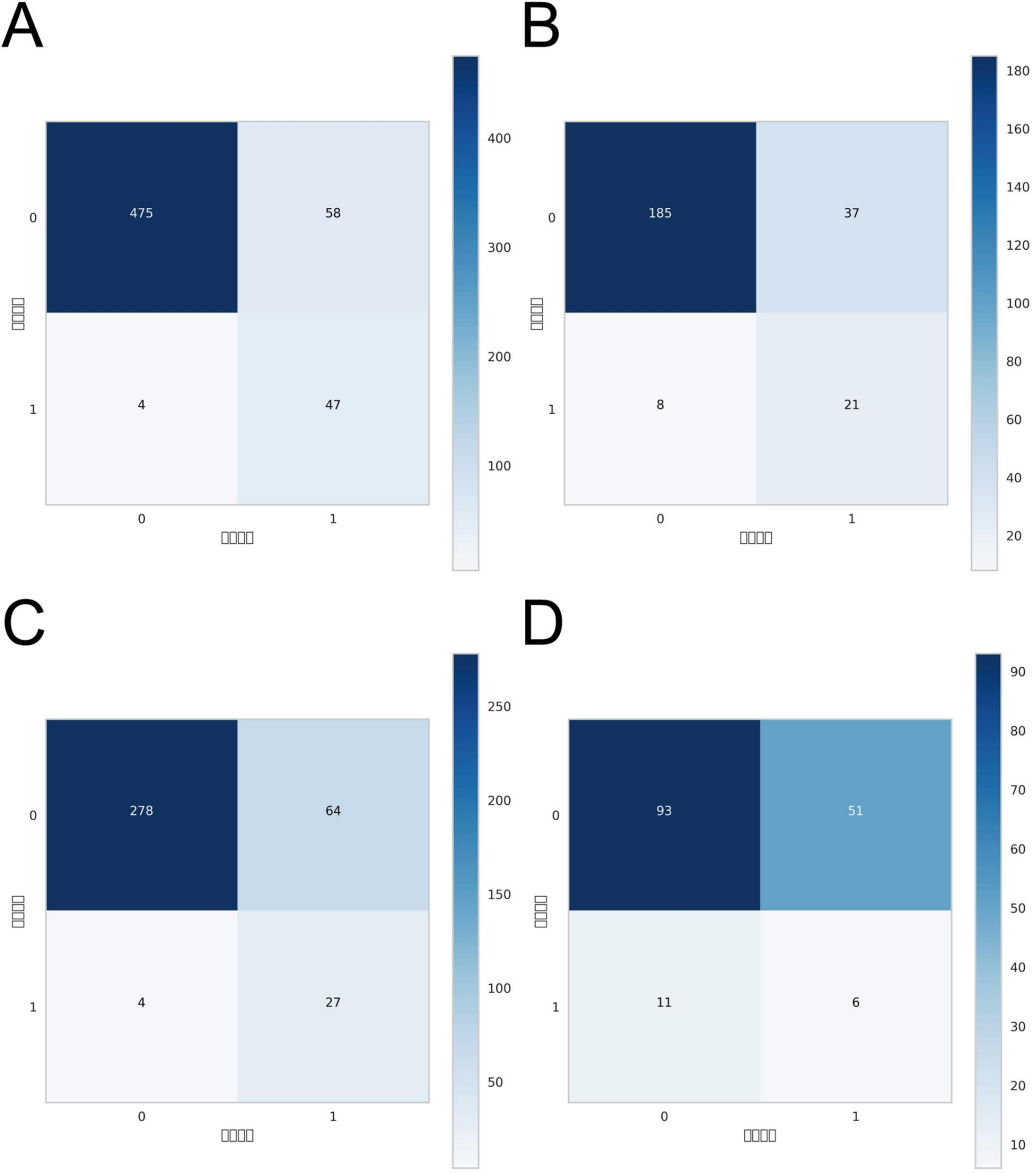
Confusion matrices of the XGBoost model across different datasets: **(A)** confusion matrix for the internal test set; **(B)** confusion matrix for the internal validation set; **(C)** confusion matrix for the external test set; **(D)** confusion matrix for the external validation set.

**Figure 6:**
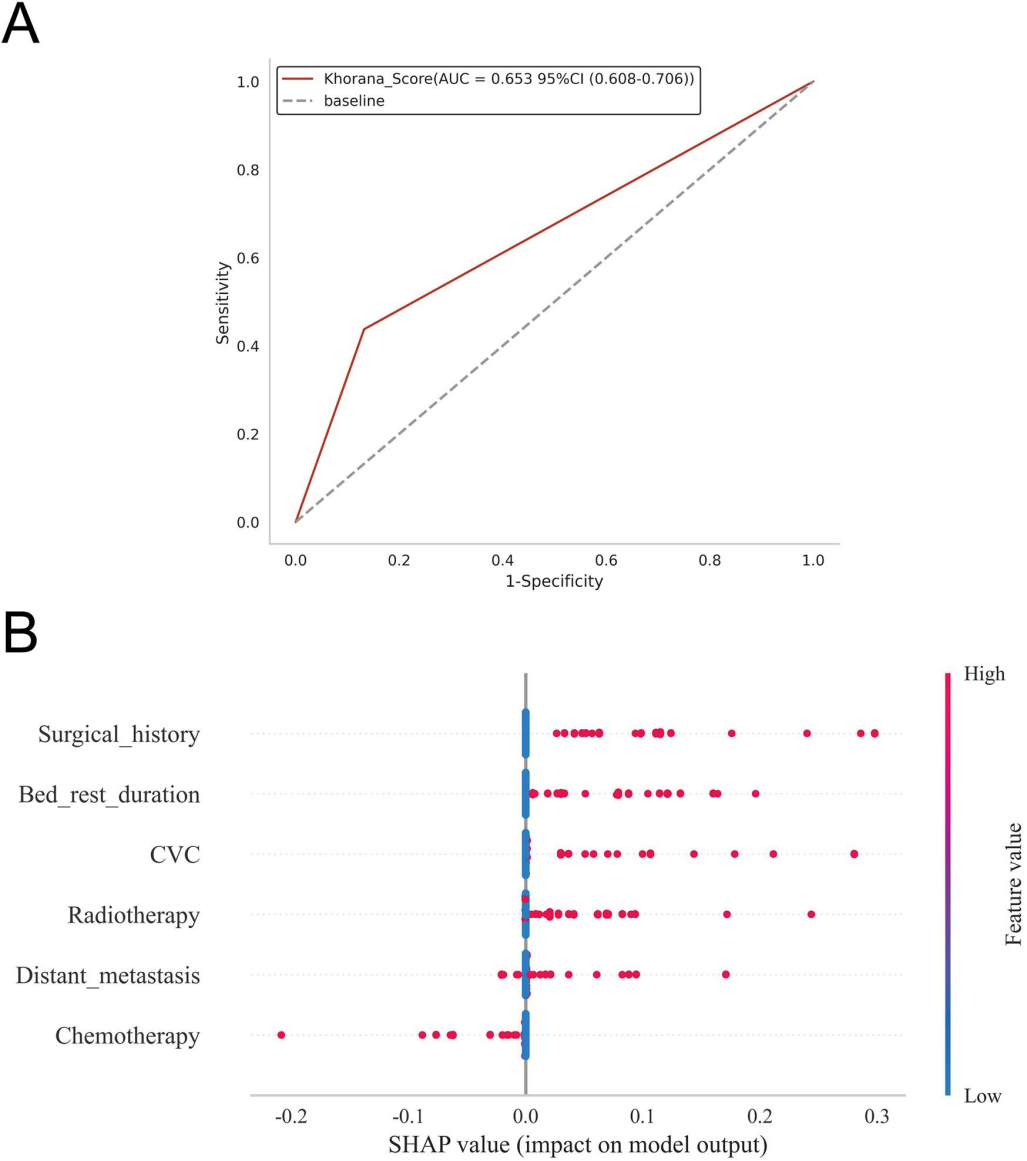
**(A)** Predictive performance of the Khorana score for thrombosis risk in the study cohort; **(B)** depicts the SHAP summary plot, ranking risk factors by their mean absolute Shapley values, with higher-ranked factors exerting a greater influence on model predictions.

ROC curve analysis demonstrated that the XGBoost model exhibited excellent predictive performance in both the training and validation sets, achieving an AUC of 0.951 in the training set and 0.882 in the validation set, surpassing the other three machine learning models (Table 3, Figures 3A,B). These high AUC values indicate outstanding discrimination ability, effectively distinguishing high-risk from low-risk patients and reflecting superior predictive accuracy. The calibration analyses revealed that the curves of all four models closely approximated the ideal 45° diagonal, signifying strong concordance between predicted risk probabilities and observed event rates, and attesting to their robust performance in probability estimation. Of particular note, the XGBoost model preserved excellent calibration across both high- and low-risk strata, accurately mirroring the true probability of DVT occurrence. Such fidelity in calibration underscores the model's reliability for individualized risk stratification in clinical settings, thereby enabling more precise preventive and therapeutic interventions. Calibration quality was further quantified using the Brier score. All four models achieved values well below 0.1 (XGBoost: 0.070; Random Forest: 0.070; SVM: 0.073; KNN: 0.065), reflecting outstanding agreement between predicted probabilities and actual outcomes. DCA demonstrated that all models—particularly XGBoost—conferred a greater net clinical benefit than the extremes of a “treat-all” or “treat-none” strategy. This advantage was most pronounced within the 0.2–0.4 risk threshold range, highlighting the models’ capacity to accurately identify high-risk patients, thereby guiding targeted thromboprophylaxis and minimizing unnecessary pharmacological interventions and their attendant adverse effects (Figures 3C,D). Notably, the XGBoost model demonstrated the greatest net benefit, underscoring its potential for precise individualized risk prediction of lower extremity DVT in patients with gastrointestinal tumors in clinical practice. To comprehensively assess model generalizability, k-fold cross-validation was performed on the internal validation set. Specifically, 125 samples (15.00%) were randomly selected as the test set, while the remainder was used for training with 10-fold cross-validation. This approach robustly evaluated model performance across diverse data subsets, minimizing bias from random splits and enhancing result reliability. During cross-validation, the XGBoost model consistently outperformed others, achieving an AUC of 0.9146 (95% CI: 0.8205–0.9934) in validation folds, an AUC of 0.8308 in the test set, and an accuracy of 0.8016 (Figures 4A–C). The RF model attained a validation AUC of 0.8029 (0.7051–0.8864), test set AUC of 0.8287, and accuracy of 0.7302. The SVM model showed a validation AUC of 0.8091 (0.6133–0.9797), but its test set AUC decreased to 0.6182 with accuracy of 0.8095. The KNN model demonstrated an AUC of 0.8240 (0.6393–0.9832) in validation, 0.7275 in the test set, and accuracy of 0.7540. Collectively, XGBoost outperformed all other models across key metrics, particularly AUC and accuracy, indicating superior discriminatory power, better generalizability, and more stable predictive performance. Consequently, XGBoost was selected as the optimal algorithm for predicting high-risk factors of lower extremity venous thrombosis in this study. In the external validation cohort, ROC analysis revealed an AUC of 0.681 (Figure 4D), demonstrating that the model maintained reasonable predictive accuracy on unseen data and exhibited satisfactory generalization capability. In this study, confusion matrices were constructed for the XGBoost model across multiple datasets. Comparative analysis of these matrices enabled a more precise evaluation of the model's propensity for false negatives and false positives in identifying patients with lower extremity DVT, thereby offering critical insights for clinical threshold optimization and risk management (Figures 5A–D). Retrospective assessment of the Khorana score revealed an AUC of 0.653 (95% CI: 0.608–0.706) within our cohort, indicative of moderate predictive capability. By contrast, the machine learning models developed herein—particularly the XGBoost model—exhibited markedly superior performance, achieving an AUC of 0.951 in the training set and 0.882 in the validation set, thereby substantially surpassing the traditional Khorana score. These elevated AUC values underscore the XGBoost model's enhanced discriminatory power and superior predictive accuracy in differentiating high-risk from low-risk patients (Figure 6A).

**Table 3:** Summarizes the performance metrics of the four predictive models evaluated in this study.

		AUC (95% CI)	Accuracy (95% CI)	Sensitivity (95% CI)	Specificity (95% CI)	F1 score (95% CI)
KNN	Training set	0.907 (0.865–0.948)	0.851 (0.826–0.877)	0.872 (0.831–0.912)	0.849 (0.818–0.880)	0.536 (0.502–0.571)
	Validation set	0.838 (0.731–0.944)	0.843 (0.818–0.868)	0.758 (0.679–0.838)	0.853 (0.820–0.886)	0.486 (0.448–0.523)
XGBoost	Training set	0.951 (0.931–0.970)	0.853 (0.839–0.867)	0.925 (0.911–0.938)	0.845 (0.829–0.861)	0.548 (0.529–0.567)
	Validation set	0.882 (0.809–0.955)	0.839 (0.823–0.855)	0.724 (0.649–0.798)	0.852 (0.831–0.874)	0.463 (0.428–0.499)
RF	Training set	0.893 (0.856–0.929)	0.871 (0.857–0.885)	0.759 (0.737–0.782)	0.883 (0.865–0.900)	0.532 (0.509–0.554)
	Validation set	0.873 (0.793–0.953)	0.864 (0.844–0.884)	0.679 (0.628–0.731)	0.884 (0.861–0.906)	0.495 (0.459–0.530)
SVM	Training set	0.859 (0.811–0.908)	0.826 (0.757–0.894)	0.769 (0.691–0.848)	0.832 (0.748–0.915)	0.494 (0.427–0.562)
	Validation set	0.780 (0.642–0.918)	0.817 (0.732–0.903)	0.644 (0.503–0.785)	0.835 (0.732–0.939)	0.437 (0.359–0.514)

CI, confidence interval.

### Model explanation

**Figure 7:**
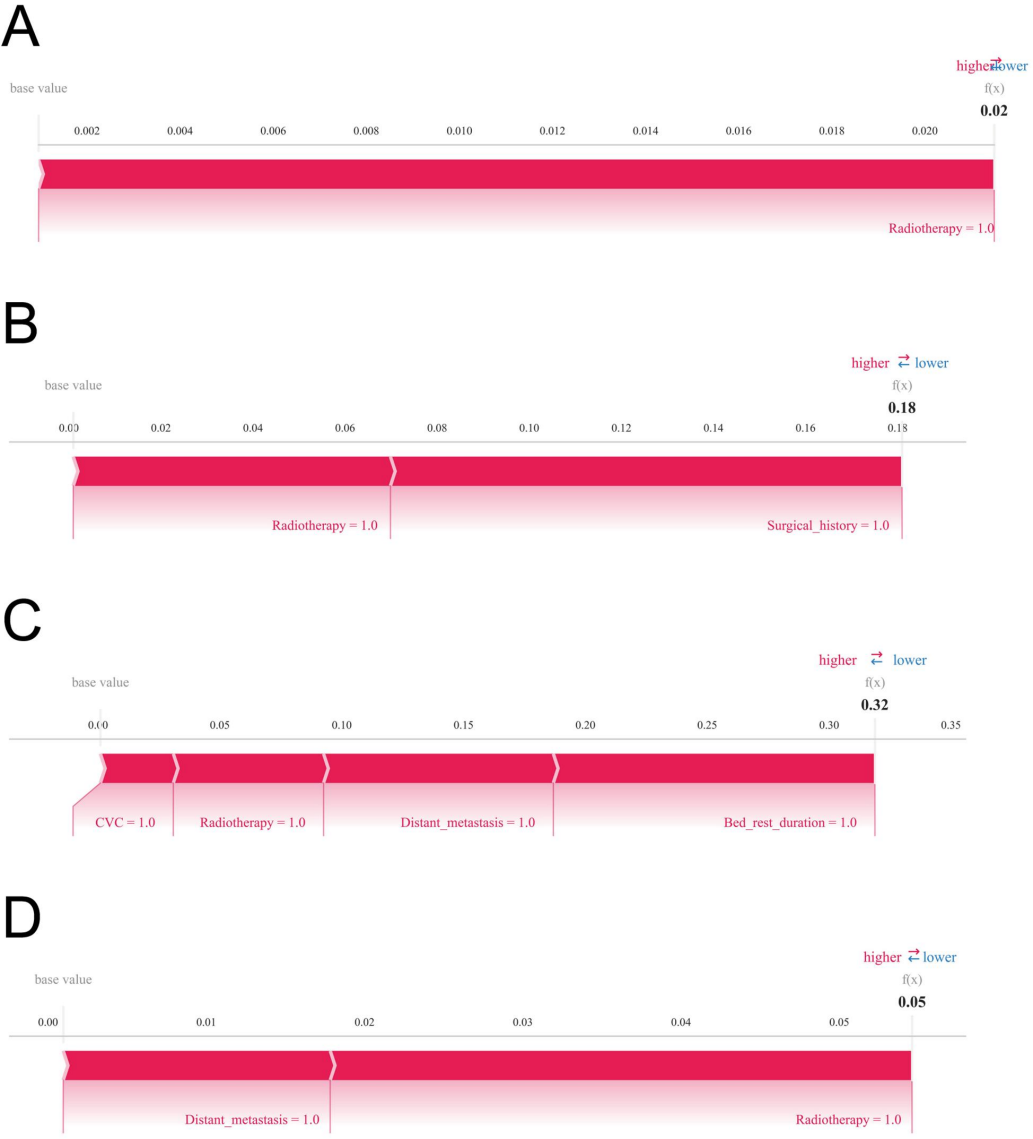
SHAP force plots are displayed to visualize individual-level explanations of the predictions. Variables are arranged horizontally according to their absolute impact, with blue indicating features that decrease predicted risk (negative SHAP values) and red indicating features that increase predicted risk (positive SHAP values). **(A)** Predictive analysis of Patient I. **(B)** Predictive analysis of Patient II. **(C)** Predictive analysis of Patient III. **(D)** Predictive analysis of Patient IV.

The SHAP summary plot (Figure 6B) highlights the primary risk factors for lower extremity venous thrombosis and their relative importance. The analysis identified surgical treatment, prolonged bed rest, central venous catheterization, radiotherapy, distant tumor metastasis, and chemotherapy as the most influential predictors. To further assess the model's clinical applicability, personalized predictions for four individual patients were examined using SHAP force plots (Figures 7A–D), which detailed the specific risk factors and their contributions for each case:

Patient 1: The model predicted a low probability of developing lower extremity venous thrombosis (0.02), with radiotherapy as the main influencing factor. Patient 2: The predicted risk was 0.18, primarily driven by radiotherapy and surgical treatment. Patient 3: The predicted probability was 0.32, reflecting a moderate risk predominantly contributed by prolonged bed rest, central venous catheterization, radiotherapy, and distant tumor metastasis. Patient 4: The model estimated a risk of 0.05, mainly influenced by radiotherapy and distant metastasis, indicating a relatively low yet clinically relevant risk warranting attention.

## Discussion

This study harnessed four widely acclaimed machine learning algorithms—XGBoost, RF, SVM, and KNN—to construct a predictive model for lower extremity DVT. Each algorithm embodies distinct strengths tailored to diverse data structures and clinical contexts (25, 26). XGBoost, an ensemble method grounded in gradient boosting, excels at managing high-dimensional data while mitigating overfitting, showcasing remarkable fitting capacity and model expressiveness. It is particularly proficient at capturing intricate nonlinear relationships and complex variable interactions. Random Forest, another ensemble approach, builds a multitude of decision trees and synthesizes their outputs via majority voting, exhibiting resilience to noise and missing data, coupled with robust generalizability. SVM, predicated on the principle of maximum margin classification, is especially potent in small-sample, high-dimensional scenarios; its kernel functions adeptly handle nonlinear and non-separable data. KNN, reliant on sample proximity for classification, is lauded for its simplicity and ease of deployment, particularly when data distribution is relatively uniform and class boundaries are distinct (27–29).

Despite the merits inherent in each algorithm, XGBoost surpassed its counterparts across our dataset. It consistently manifested superior discriminatory power in both training and validation cohorts, adeptly distinguishing between high- and low-risk patients. Calibration analyses demonstrated a remarkable concordance between predicted probabilities and observed outcomes, with calibration curves nearly coinciding with ideal reference lines, reflecting precise risk estimation. Moreover, XGBoost sustained elevated predictive accuracy following cross-validation and external validation, underscoring its robustness and translational viability. Decision curve analysis further accentuated its superior net clinical benefit across diverse risk thresholds, reinforcing its utility in clinical decision-making. Conversely, the alternative models exhibited certain limitations: Random Forest, while stable during training, displayed modest declines in validation accuracy and was hindered by complexity and sensitivity to feature redundancy, adversely affecting discrimination. SVM achieved commendable training accuracy but suffered a marked drop in test performance, indicative of overfitting; its computational intensity also restricts scalability with larger datasets or numerous variables. KNN's test set performance was moderate yet susceptible to uneven sample distribution and noise, resulting in instability; its efficacy is further compromised by sensitivity to feature scaling and dependence on meticulous preprocessing. Considering a spectrum of evaluation metrics and overarching model performance, XGBoost was ultimately adjudged the optimal algorithm for predicting lower limb DVT risk.

In comparison to conventional diagnostic paradigms, the XGBoost-based machine learning model developed herein exhibits marked superiority in performance and clinical applicability across multiple facets. Traditional risk prediction methodologies often hinge upon presupposed linear associations and assumptions of variable independence, thereby constraining their capacity to unveil latent nonlinear structures and the intricate interplay of variables intrinsic to high-dimensional clinical datasets. Consequently, such approaches are frequently limited in accuracy, generalizability, and adaptability within clinical contexts. In this study, a supplementary retrospective analysis was undertaken to evaluate the predictive performance of the Khorana score for thrombosis risk within the study population. The Khorana score yielded an AUC of 0.653 (95% CI: 0.608–0.706), notably lower than that achieved by the machine learning models developed herein, such as XGBoost, thereby highlighting a discernible gap in predictive accuracy. Consistent with our findings, Mulder et al. reported that, among outpatient cancer patients, only 23.4% (95% CI: 18.4%–29.4%) of those who developed VTE were classified as high risk by the Khorana score (30). While the Khorana score remains a useful tool for identifying high-risk patients and informing thromboprophylaxis, the majority of thrombotic events occur in individuals categorized as non–high risk. Such limitations underscore the restricted predictive capacity of traditional risk assessment methods, particularly in the context of certain tumor types and interindividual variability. By contrast, XGBoost, as a gradient-boosting ensemble algorithm, affords exceptional feature representation, resilience to noise, and robustness against missing data, enabling nuanced modeling of complex clinical phenomena and yielding refined, stable individualized risk estimations (31–33).

To augment interpretability and practical utility, we integrated SHAP analysis to systematically deconstruct the predictive framework of the XGBoost model. Rooted in cooperative game theory, SHAP quantifies the marginal contribution of each clinical variable to model predictions in a consistent and locally faithful manner, thereby facilitating personalized risk elucidations for individual patients. This innovation not only enhances transparency and interpretability but also equips clinicians with lucid, actionable insights that bolster confidence and encourage pragmatic adoption of model-assisted decision-making in routine care (34, 35). The SHAP analysis pinpointed surgery, prolonged immobilization, central venous catheterization, radiotherapy, distant tumor metastasis, and chemotherapy as the foremost clinical determinants of lower limb DVT risk. These features manifested pronounced importance within the model, underscoring their plausible pathophysiological roles in thrombogenesis and highlighting their priority in perioperative risk stratification and targeted intervention. Clinically, the model enables early identification of high-risk patients in both pre- and postoperative settings, optimizing anticoagulation strategies and mitigating DVT incidence, thereby refining overall perioperative management. From the patient perspective, personalized risk interpretations foster heightened awareness and engagement, advancing the paradigm of patient-centered precision medicine.

The canonical Virchow's triad—comprising hemodynamic alterations (venous stasis), endothelial injury, and hypercoagulability—remains the foundational framework for understanding venous thromboembolism pathophysiology (22, 36, 37). Our machine learning findings resonate with this model, as the identified risk factors—including surgical intervention, prolonged bed rest, central venous catheterization, radiotherapy, distant metastasis, and chemotherapy—correspond intimately with these core pathological processes. Surgical procedures, by virtue of their invasiveness, induce direct endothelial trauma, precipitating localized inflammatory cascades and endothelial dysfunction that compromise anticoagulant defenses, thereby fostering thrombogenesis. Additionally, perioperative immobilization impairs the efficacy of the muscular pump, precipitating venous stasis. The systemic inflammatory milieu and stress response elicited by surgery further amplify hypercoagulability, collectively orchestrating thrombus formation via multifaceted synergistic pathways (38, 39). Prolonged immobilization curtails lower limb muscular contractions, diminishing venous return and exacerbating blood flow stasis, which prolongs blood constituent interactions and cultivates hypoxic microenvironments that activate endothelial cells and upregulate procoagulant factors, thereby potentiating hypercoagulability. Central venous catheterization, a ubiquitous clinical intervention, disrupts endothelial integrity mechanically and triggers local coagulation cascades alongside inflammatory responses. Turbulence and stasis associated with catheter placement, compounded by infection and inflammation, exacerbate endothelial dysfunction and hypercoagulable states (40, 41). Radiotherapy inflicts direct cytotoxicity upon endothelial cells, undermining structural integrity and anticoagulant functionality while inducing procoagulant and inflammatory mediator expression, generating a localized prothrombotic milieu. Radiation-induced fibrosis and vascular stenosis further perturb hemodynamics, promoting stasis (42–44). The presence of distant tumor metastases signifies an elevated tumor burden and systemic disease progression; metastatic cells secrete procoagulant agents (e.g., tissue factor, cytokines) that systemically activate coagulation pathways, markedly intensifying hypercoagulability. Concurrent chronic inflammation and immune dysregulation erode endothelial integrity and facilitate platelet activation and fibrin deposition, fostering a thrombogenic microenvironment (45, 46). Chemotherapeutic agents exert direct endothelial toxicity, impairing cellular architecture and function, while suppressing hematopoiesis and immune surveillance, heightening susceptibility to infection and secondary endothelial inflammation. Certain chemotherapies modulate platelet activity and blood rheology, thereby contributing to venous stasis and hypercoagulability, cumulatively elevating thrombotic risk. Collectively, these delineated risk factors converge upon the pillars of Virchow's triad, driving the pathogenesis of lower limb DVT through interdependent mechanisms of stasis, endothelial injury, and hypercoagulability.

Previous studies (15, 16) have proposed that the type of gastrointestinal malignancy—such as gastric, colorectal, or esophageal cancer—may modulate the risk of lower limb DVT through variations in tumor biology, anatomical location, and treatment approaches. However, our analysis did not reveal a significant correlation between tumor type and DVT incidence, a discrepancy attributable to several factors. From a mechanistic standpoint, DVT pathogenesis fundamentally revolves around Virchow's triad, which remains largely consistent across different gastrointestinal cancers. Irrespective of tumor origin, advanced malignancy is commonly accompanied by shared clinical factors including prolonged immobilization, surgical trauma, central venous catheterization, chemotherapy, and radiotherapy, all of which activate thrombogenic pathways in a similar manner across tumor types. Consequently, these ubiquitous risk factors may eclipse any potential tumor site-specific influences. Additionally, our model prioritized actual clinical interventions and functional status variables (e.g., surgery, chemoradiotherapy, immobilization) over tumor classification, resulting in greater weighting of treatment-related predictors relative to tumor location in multivariate analyses. Finally, machine learning algorithms inherently focus on variables that optimize predictive performance; thus, tumor type, despite possible biological relevance within certain subsets, conferred limited incremental predictive value and was consequently assigned lower importance and excluded from key predictors.

A pronounced disparity in AUC performance was observed for the XGBoost model between the internal validation cohort and the external test cohort. Given that patients from different hospitals were enrolled contemporaneously, the influence of temporal factors on model performance is likely negligible. This divergence is chiefly attributable to inter-hospital heterogeneity in patient demographics, disease severity, comorbidities, and treatment regimens, which engenders distributional shifts within the external dataset. Furthermore, inconsistencies in clinical testing methodologies, data recording standards, and laboratory procedures across institutions may compromise the uniformity and quality of input variables, thereby constraining the model's generalizability. Variations in sample size and the prevalence of DVT events within the external validation cohort may also contribute to performance variability. Notably, the implementation of 10-fold cross-validation and regularization techniques in this study effectively mitigated overfitting risks, bolstering model robustness and generalizability, and highlighting the rigor of our training methodology.

This study presents several strengths in forecasting lower limb DVT risk. The utilization of a large, multidimensional clinical dataset—including surgical treatment, immobilization status, central venous catheterization, oncologic therapies, and metastatic burden—enhances the model's representativeness and clinical relevance. A rigorous comparison of four prominent machine learning algorithms facilitated the identification of XGBoost as the superior method, demonstrating consistent excellence in discrimination, calibration, and clinical utility across training, internal validation, and external validation cohorts. The incorporation of SHAP analysis further enriched interpretability, fostering clinical confidence and easing model integration into practice.

Nonetheless, this study is subject to several limitations. We observed an inverse association between chemotherapy and the risk of lower extremity deep vein thrombosis (DVT), a finding that diverges from conventional clinical understanding and likely reflects the interplay of multiple factors rather than a direct protective effect of chemotherapy itself. First, as a retrospective investigation, reliance on historical clinical records may introduce incomplete data, recording biases, and inconsistencies in variable definitions, potentially compromising model accuracy. Although 34 clinical variables were incorporated and feature selection was conducted through multivariate regression and diverse machine learning algorithms, residual confounding—such as anticoagulant use, specific chemotherapy regimens, and patients’ nutritional and activity status—may persist. Moreover, patients eligible for chemotherapy generally exhibit superior overall health and physiological reserve, whereas those not receiving treatment often present with more severe disease or comorbidities, conferring higher intrinsic thrombotic risk. Additionally, patients undergoing chemotherapy are frequently managed within tertiary care centers, benefiting from structured perioperative assessment and thromboprophylactic protocols, which may further mitigate thrombotic events. Collectively, these observations suggest that the relationship between chemotherapy and thrombotic risk is more nuanced than traditionally perceived, warranting further exploration in larger, prospective studies incorporating detailed therapeutic and management data. In this study, we implemented 10-fold cross-validation and incorporated an external validation cohort to attenuate the risk of model overfitting. Nonetheless, the relatively limited sample size imposes intrinsic constraints, leaving residual concerns regarding potential overfitting. Furthermore, the model's sensitivity, F1 score, and external validation outcomes suggest that its clinical utility for the early identification of high-risk patients remains somewhat circumscribed. Future investigations will aim to substantiate the model's generalizability and practical applicability through validation in larger, prospective cohorts. To enhance the transparency and interpretability of our machine learning framework, we applied the SHAP methodology. SHAP rigorously quantifies the individual contribution of each feature to model predictions, thereby elucidating the decision-making process and fostering clinician trust and acceptance. However, despite offering valuable local interpretability, SHAP and analogous *post hoc* explanation tools remain inherently complementary to black-box models and possess intrinsic constraints. Machine learning algorithms, particularly those employing deep learning architectures, continue to be perceived as opaque “black boxes” due to their complexity and inscrutable internal mechanics (47, 48). This opacity may undermine clinical confidence in model outputs, impeding their translation into routine medical practice. Accordingly, advancing model interpretability is imperative to facilitate broader acceptance and practical deployment in clinical settings. Future research should prioritize the development of inherently transparent and interpretable model architectures to bolster the reliability and efficacy of clinical applications. Moreover, certain potential risk factors—such as genetic predispositions, molecular biomarkers, and lifestyle factors—were not comprehensively included, indicating avenues for future inquiry. In this study, the prevalence of DVT was approximately 9.35%, reflecting a notable class imbalance. Although techniques such as SMOTE, undersampling, or class weighting were not employed, the inherent robustness of XGBoost and Random Forest models to imbalanced data mitigated some related challenges. Furthermore, the use of stratified sampling in conjunction with 10-fold cross-validation enhanced model stability and generalizability. The lack of dedicated imbalance correction methods may have compromised the performance of models such as SVM and KNN in accurately identifying minority class instances, representing a limitation of this study. Future investigations will explore the integration of SMOTE, class weighting, and other approaches to systematically assess their influence on model efficacy.

In summary, the XGBoost-based machine learning model developed herein constitutes a powerful, interpretable, and clinically actionable tool for individualized prediction of lower extremity DVT risk among patients with gastrointestinal malignancies. For patients identified as high risk, we provide clear delineation of personalized thrombosis probabilities alongside the principal contributory factors, thereby fostering patient comprehension of risk magnitude while emphasizing that this represents a risk stratification rather than a diagnostic conclusion—ultimately facilitating early prevention and management. Looking ahead, we intend to embed the XGBoost prediction model within electronic medical record (EMR) systems to enable real-time risk assessment and alerting of high-risk individuals, thereby empowering clinicians to devise precise, tailored prophylactic strategies. By elucidating perioperative and oncologic risk determinants within the conceptual framework of Virchow's triad, this model holds substantial promise for refining perioperative risk stratification, guiding targeted preventive interventions, and ultimately enhancing patient outcomes through precision medicine.

## Conclusion

This study rigorously assessed the predictive capabilities of four leading machine learning algorithms for lower extremity DVT risk, grounded in multidimensional clinical data, ultimately designating XGBoost as the superior model. Leveraging SHAP analysis, the model affords individualized interpretability of its predictions. It exhibited exceptional discriminatory accuracy in stratifying high- vs. low-risk patients, coupled with robust generalizability, stability, and marked utility in clinical decision-making. Importantly, the model demonstrates substantial translational potential in postoperative management, oncologic risk evaluation, and tailored thromboprophylaxis. Moreover, the investigation elucidated surgery, prolonged immobilization, central venous catheterization, radiotherapy, distant tumor metastasis, and chemotherapy as pivotal contributors to DVT pathogenesis, thereby enriching the mechanistic insight into thrombogenesis.

## Data Availability

The original contributions presented in the study are included in the article/Supplementary Material, further inquiries can be directed to the corresponding authors.
